# Extraction of Cyclic Oligomer and Their Influence on Polyester Dyeing in a Silicone Waterless Dyeing System

**DOI:** 10.3390/polym13213687

**Published:** 2021-10-26

**Authors:** Hao Li, Liujun Pei, Hongjuan Zhang, Zhiwen Wang, Muhammad Asad Saleem, Omer Kamal Alebeid, Jiping Wang

**Affiliations:** 1 Engineering Research Center of Textile Chemistry and Clean Production, Shanghai University of Engineering Science, Shanghai 201620, China; whoarethere@163.com (H.L.); zhjdhdx@163.com (H.Z.); 17805051455@163.com (Z.W.); 2 School of Textiles and Fashion, Shanghai University of Engineering Science, Shanghai 201620, China; 3 School of Engineering Research Center for Eco-Dyeing and Finishing of Textiles, Zhejiang Sci-Tech University, Hangzhou 310018, China; asadsaleem92@yahoo.com (M.A.S.); omer_z22@hotmail.com (O.K.A.)

**Keywords:** polyester, oligomer, waterless dyeing, DSC, DMA, XRD

## Abstract

As a promising new dyeing process without using water, the non-aqueous medium dyeing of polyester has attracted people’s attention and some progress has been made in related research. However, the oligomers of polyester fiber can affect the dyeing of polyester during the use of a silicone waterless dyeing system. Based on this point, the oligomer problem in the silicone waterless dyeing system was investigated. The oligomers of some different types of polyester were extracted by solvent extraction. A treatment method with little influence on the fiber was used to reduce the oligomer content in polyester. The improvement of the dyeing effect of polyester after treatment in silicone medium was studied, and the influence of the oligomer on polyester dyeing was also analyzed. For the dyeing of disperse blue 366, the dye exhaustion was increased by 3.25–3.71%, and the color depth of the dyed sample was increased by 6–13%. Moreover, the colorfastness to rubbing was also improved. In the comparison, the changes in thermal properties and crystallization properties of polyester were tested by differential scanning calorimetry (DSC), dynamic mechanical analysis (DMA), and X-ray diffraction analysis (XRD). The results showed that the thermal and crystalline properties of polyester fiber were not changed before and after solvent extraction.

## 1. Introduction

As is well known, disperse dyes are usually employed to dye PET fabrics in a traditional water bath. As the solubility of disperse dyes in water is usually very low, it is necessary to use high-temperature and high-pressure equipment for dyeing [1]. Furthermore, a large amount of water, dispersants, and other additives are used during dyeing [2], the result of which is a production process that consumes a lot of water resources, and brings great challenges to wastewater treatment [3,4]. In view of this, many new water-saving and waterless dyeing processes have been developed. Alya et al. reported a method of dyeing that utilizes a carrier. In this process, enough dye is dispersed to the interface of fiber and water [5]. A supercritical carbon dioxide (CO_2_) dyeing process can effectively solve a series of wastewater discharge problems [6]. Not only is the dyeing time short, but excellent dyeing effects can also be achieved [7]. This dyeing method makes it possible to recycle undyed disperse dyes [8]. However, supercritical carbon dioxide (CO_2_) needs to be maintained under high pressure (>7.38 MPa), which requires high equipment safety. It may be one of the technologies that will change the development of the industry.

The silicone waterless dyeing system is an excellent representative of such systems [9,10]. The silicone waterless dyeing system does not need to use any dispersant and leveling agent. After dyeing, the solubility of disperse dyes in silicone solvent is relatively low at room temperature [11]; therefore, disperse dye and dyeing media can be easily separated by static filtration after the residual dyebath is cooled down. Therefore, silicone solvent can be recovered or recycled as a dyeing medium to achieve sustainable dyeing [12].

At present, there are two main problems in the dyeing of polyester in the silicone waterless dyeing system. Firstly, it is necessary to select some disperse dyes with low solubility in the silicone medium [13], which is conducive to the adsorption of dissolved dyes in the open macromolecular chain segments during dyeing. Some dye-promoting agents are considered to decrease the solubility of dye in silicone solvent, improving the final uptake of dye [14,15]. Yu et al. [11] improved the dyeing effect of disperse dyes by synthesizing new dyes with appropriate solubility in silicone waterless dyeing media. Wang et al. [12] showed that some dye-promoting agents can also improve the dyeing effect of polyester fabric. Pei et al. [10] also confirmed that the use of dispersants did greatly improve the uptake of dye and the color depth of dyed samples. However, such work will undoubtedly increase difficulties in the recovery of dyeing media. As with traditional water bath dyeing, a certain amount of small molecular oligomers will be precipitated with the opening of polyester macromolecular segments in the dyeing process and dispersed in the dyeing solution at higher temperature. However, due to the low solubility of oligomers in the dyeing system, these oligomers crystallize and adsorb on the dyeing equipment and even the fabric surface during the cooling process after dyeing, which greatly affects the dyeing fastness and the color difference of the dyed fabric. 

The content of PET cyclic trimer is higher than that of other oligomers because of its high chemical and thermal stability and easy aggregation and crystallization during dyeing [16,17]. Not only is the melting point of cyclic trimer as high as 310 °C [18], its solubility in water is also very low [19]. Therefore, cyclic trimer is difficult to remove in polyester pretreatment [20], dyeing or traditional wet treatment processes, which affects the dyeing process of polyester. In a traditional water bath dyeing system, reduction cleaning is usually used to wash away loose dyes and oligomers on the fiber surface during dyeing. Montero et al. [20] reported the problems caused by the precipitation and deposition of oligomer during SC-CO_2_ dyeing, and predicted the possible effects of temperature, flow rate and pressure. However, how to remove polyester oligomer during the dyeing process in silicone waterless dyeing systems is rarely reported, which will hinder the development of this ecological dyeing technology. 

In this investigation, the improvement of dye uptake and leveling property of polyester by extracting the surface oligomer on PET fabric was studied. The differential scanning calorimetry (DSC), dynamic mechanical analysis (DMA) and X-ray diffraction (XRD) of the polyester before and after oligomer extraction were studied. The improvement of the fabric dyeing effect was also measured. According to the research results and the analysis of the performance characteristics of oligomers, the key dyeing effect evaluation index is eduction of the oligomer in the silicone waterless dyeing system, which can provide the direction for using effective methods to remove oligomers for subsequent research on improving the dyeing effect of polyester silicone waterless dyeing media.

## 2. Materials and Methods

### 2.1. Materials

Polyester yarns and fabric (100% PET) were purchased from Zhejiang Tongkun Group (Jiaxing, China). Disperse Blue C.I. 366 (filter cake, no dispersant) and D5 (purity 98%) were provided by Zhejiang Green Universe Textile Technology Co., Ltd. (Jiaxing, China). Tetrachloroethane (analytical purity) was purchased from Fisher Scientific (Bergen County, NJ, USA). DMSO (dimethyl sulfoxide, analytical purity) was purchased from Tianjin Kemiou Chemical Reagent Co., Ltd. (Tianjin, China). Soap Slice (industrial grade) was provided by mien testing instrument (Shanghai, China) Co., Ltd.

### 2.2. Oligomer Treatment Method and Dyeing of Polyester Fabric

#### 2.2.1. Quantitative Analysis of Oligomers

A simple and rapid method for oligomer extraction was applied in this study to directly reflect the oligomer content in the sample. Firstly, 5 g of polyester sample was added into tetrachloroethane solution at a liquor ratio of 20:1 when the temperature was 25 °C. Then, the temperature was raised to 70 °C at a rate of 2 °C/min. After 2 h, the temperature was reduced to room temperature (25 °C) at a rate of 0.5 °C/min. After filtration, the solution was rotary-evaporated to obtain a concentrated solution at 50 °C when the vacuum was 35 mmHg. The concentrated solution was naturally dried in a container, and the mass of the white powder was weighed. According to the mass of the sample extracted, the oligomer content of the polyester (yarn or fabric) was calculated. The formula for calculating oligomer content is as Equation (1).

(1) $$C_{0}=\frac{m_{a}-m_{b}}{m_{0}}\times 100\%$$

where *C*_0_ is the oligomer content, *m_a_* is the weight of the container after drying the concentrated solution, *m_b_* is the dry weight of the container before pouring the concentrated solution, and *m*_0_ is the quality of the polyester sample before extraction.

#### 2.2.2. Pretreatment of Polyester Sample

To investigate the influence of oligomer on polyester dyeing properties, polyester was treated with tetrachloroethane in a condensation reflux device at 35 °C for 40 min. The bath ratio of polyester fabric to tetrachloroethane was 20:1. After treating, these solutions were used to determine the content of oligomers by HPLC method, and the polyester fabric was dried at 80 °C for 60 min. Then, the polyester fabric was dyed in the silicone waterless dyeing system.

#### 2.2.3. High-Performance Liquid Chromatography (HPLC) Analysis

A Waters e2695 liquid chromatograph (Waters, Milford, MA, USA) equipped with a Waters 2998 HPLC PDA photodiode matrix detector was employed to analyze oligomers. An XBridge C18 chromatographic column (5 μm, 4.6 × 250 mm^2^) was used for separation. Samples (1 mg/ mL) were prepared in tetrachloroethane and injected via an autosampler. The injection volume was 20 μL, and the running time was 21 min at a flow rate of 1 mL/min. The maximum absorption wavelength of the oligomer was determined by an ultraviolet–visible spectrophotometer at 247 nm (according to the UV measurement results). Before analysis, the oligomer solution was filtered with a 0.22 μm organic phase syringe filter. The gradient mode used is shown in Table 1.

#### 2.2.4. Dyeing of Polyester in the Silicone Waterless Dyeing System

Polyester samples weighing 5 g were dyed with 2.0% o.w.f. (on weight of fabric) of disperse dye in the silicone waterless dyeing system at a liquor ratio of 20:1. Dyeing was performed at 25 °C for 10 min, after which the temperature was raised to 80 °C at a rate of 6 °C/min. After 10 min, the temperature was raised to 140 °C at a rate of 3 °C/min and maintained for 60 min. After dyeing, the temperature was dropped to 80 °C. Finally, the dyed samples were washed twice using D5 at a liquor ratio of 20:1 at 80 °C for 15 min.

### 2.3. Characterization and Test Methods

#### 2.3.1. Exhaustion Percent

In the dyeing of soluble dyes, the dye uptake can be determined by measuring the absorbance before and after dyeing. The dye uptake was determined by measuring the absorbance of residual liquid at the maximum absorption wavelength. In this solvent dyeing process, a high proportion of dyes in the dyeing residue are not actually dissolved. In order to make the test results accurate, DMSO (dimethyl sulfoxide) was used to extract dyes from the dyeing system for determination. Then, the absorbance of DMSO solution was measured. The dye exhaustion was calculated by using Equation (2).

(2) $$E\%=\;\left(1-\frac{CV}{m}\right)\times \;100\%$$

where *E* refers to the dye exhaustion, *C* refers to the dye concentration of DMSO-extracted dyes (g/L), *V* refers to the volume of DMSO (L), and *m* refers to the total dye amount added initially in the dyeing bath (g).

#### 2.3.2. Color Strength

The color strength (*K*/*S* value) was analyzed to evaluate the depth of dyed samples. A Datacolor 800 (Datacolor, NJ, USA) spectrophotometer was employed to measure the *K*/*S* value of dyed fabrics. The color testing was performed at 10° from the observer with D65 illuminant.

#### 2.3.3. Color Uniformity

The color uniformity (ΔE) of dyed polyester fabric is an important factor to illustrate the uniformity of dyeing. For each dyed fabric, 12 different points were randomly selected to measure the *K*/*S* value [21]. The calculation of color uniformity is as shown in Equations (3) and (4).

(3) $$S=\sqrt{\frac{\sum_{i=1}^{n}{\left(K/S_{i}-\bar{K/S}\right)}^{2}}{n-1}}$$

(4) $$CV\%=\frac{S}{K/S}\times 100\%$$

where *S* is the standard deviation, $\bar{K/S}$ is the average value of *K/S* values at 12 points, *CV* is the coefficient of variation, and *n* is the total number of test points. Here, we use the deviation of color depth to evaluate levelness. The smaller the *CV* value, the better the color uniformity of the fabric.

#### 2.3.4. Differential Scanning Calorimetry (DSC) Characterization

The DSC test was conducted with a DSC4000 differential scanning calorimeter produced by PerkinElmer Co., Waltham, MA, USA; a 6 mg of sample was assessed in an aluminum crucible. The test temperature was set at the heating rate of 10 °C/min, and the temperature range was warmed up to 500 °C from 40 °C. The test was carried out under a nitrogen atmosphere.

#### 2.3.5. Dynamic Mechanical Analysis (DMA)

To study the thermomechanical properties of fabrics, dynamic mechanical analysis (DMA) was performed by a Q850 dynamic thermomechanical analyzer (TA Instruments, New Castle, DE, USA) using parallel plate compression. This test was carried out to determine the storage modulus (E’), dumping factor (tan δ), melting temperature (Tm), and glass transition temperature (Tg) of the samples. Measurements were taken from 25 to 250 °C at a 3 °C/min heating rate and a 1 Hz fixed frequency. Samples of 0.15 × 5 × 80 mm^3^ in size were measured in air using a single cantilever mode.

#### 2.3.6. X-ray Diffraction Analysis (XRD) Analysis

X-ray diffraction analysis was performed using a rotating copper anode Rotaflex Smart Lab 9 kw (Rigaku, Tokyo, Japan). Settings of the measurements were the following: the scanning angle range was 5–90°, the scanning speed was 2°/min, and the ray target was copper. The uniformly selected scanning surface was the plane of the cloth sample to be measured.

#### 2.3.7. Colorfastness

The rubbing fastness of dyed polyester fabrics was analyzed on a Crockmaster M670 rubbing fastness tester (James H. Heal & Co., Ltd., Halifax, UK) according to ISO 105-X12:2001 method.

#### 2.3.8. Scanning Electron Microscopy Analysis

A ZEISS GeminiSEM 500 (Carl Zeiss, Oberkochen, Germany) model scanning electron microscope was used for observing surfaces of the fibers. The fibers had been metallized with gold.

## 3. Results and Discussion

### 3.1. Oligomer Content in Different Samples

In order to analyze the relationship between oligomer content and polyester fiber parameters, several polyester yarns were selected for study. It could be inferred that there is no obvious correlation between the oligomer content of polyester and the fineness of yarn. As shown in Table 2, the oligomer content of polyester filaments is 2.03% and 2.95%, respectively. The oligomer content of spun yarn used in this investigation was below 1.83%, which is usually lower than the oligomer content of the filament. The content of yarn oligomers may be related to the yarn properties because the oligomer content of polyester filament is high. Moreover, the oligomer content is more likely to be mainly related to the spinning process. According to the existing research, PET oligomers are often produced in the synthesis process [22,23]. The melting point of oligomers is higher than that of PET components [20]. As the PET melt is cooled to spin, the oligomer component with a higher supercooling degree in PET would crystallize first, and then some oligomer crystallites would become the nucleating agent of PET. Moreover, the spinning speed is positively correlated with the grain size, which will affect the ratio of crystalline region to amorphous region [24]. This can serve as a judgment that the spinning process will have a greater impact on the oligomer content. The results can also be used to determine the total content of polymers in polyester.

### 3.2. High-Performance Liquid Chromatography (HPLC) Analysis

**Figure 1:**
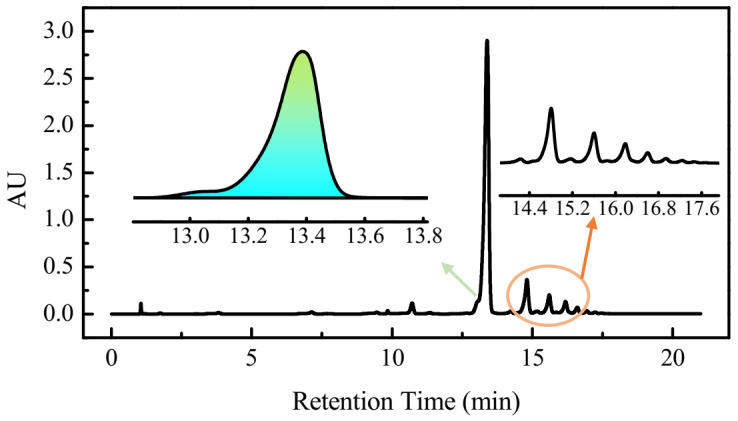
Chromatographic separation of oligomers.

In our previous studies, it has been investigated that the peaks of oligomers in liquid chromatography can be analyzed. Oligomers with different structures are separated mainly due to the size of molecular weight and their polarity. As seen in Figure 1, five different peaks were observed at t_R_ = 13.40 min, t_R_ = 14.80 min, t_R_ = 15.62 min, t_R_ = 16.19 min, and t_R_ = 16.60 min, which correspond to cyclic oligomers with different structures. Existing results indicate that the main component of the oligomer is a cyclic trimer (at t_R_ = 13.40 min). The peak area of the cyclic trimer was calculated with Waters Empower software, which accounted for about 82% of the total oligomers. The concentration was estimated from the peak area of the trimer using the calibration curve of each sample (Figure 1). With the standard curve of the cyclic trimer, we obtained the oligomer concentration in the solution of the treated polyester sample. According to this result, we found 22.56% of oligomers on the polyester were eliminated by the treatment.

### 3.3. Influence of Oligomers on the Dyeing Properties of Polyester

**Figure 2:**
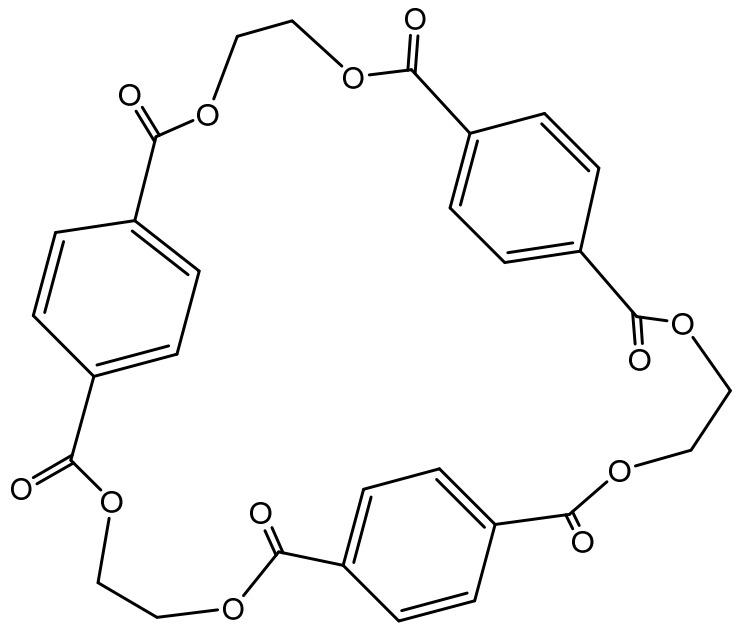
Molecular structure of cyclic trimer.

The improvement of the dyeing effects of polyester cloth after extracting oligomers was measured. The influence of oligomers on polyester dyeing is mainly evaluated from dye uptake, rubbing fastness, and levelness. Based on some previous studies, it can be concluded that most of the oligomers precipitated during dyeing are cyclic trimers; the structure is shown in Figure 2. Due to the low solubility in the dyeing medium, oligomers will aggregate in the dyeing bath and adsorb on a dyeing machine. Moreover, some oligomers will adhere to the fabric surface to form color spots, which will affect the rubbing fastness and levelness of the fabric.

**Figure 3:**
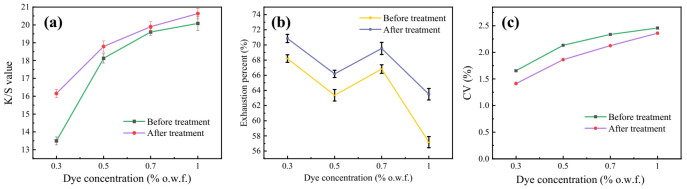
Exhaustion percent (**a**) of dye, *K*/*S* value (**b**) and color uniformity (**c**) CV% of dyed fabrics before and after extraction.

As seen in Figure 3a,b, the exhaustion of dye and the *K*/*S* value of the dyed fabric were 68.20% and 13.49, respectively, when the amount of dye concentration was 0.3% (o.w.f.). After oligomer extraction, the exhaustion of dye and the *K*/*S* value of the dyed fabric were 70.86% and 16.15, respectively. At a higher dye concentration, the effect of oligomers on dyeing depth and dye uptake has a similar trend. From these experimental results, washing away the oligomer on the surface of the polyester cloth sample has a certain effect on improving the dyeing effect. The removal of oligomers increased the dye uptake of disperse blue 366 by 3.25–3.71%. It is possible that the oligomer adsorbs some dyes, which reduces the amount of dye that the fabric can adsorb. From the *K*/*S* value of the dyed samples, the dyeing depth of cloth samples increased by 6–13% after oligomer extraction. The improvement effect is relatively good at low dye concentration. Therefore, the uptake of dye and the dyeing color depth have been improved after oligomer extraction. Obviously, the color uniformity of dyed polyester fabrics became better with the treatment in Figure 3c.

For the colorfastness to rubbing of fabrics, the oligomer treatment can reduce surface floating color. The improvement of rubbing fastness is also needed to improve the quality of fabrics in actual production. Table 3 shows the rubbing fastness of dyed fabrics before and after oligomer extraction. Before treatment, the dry rubbing fastness of dyed sample was about 4/5~2/3 level when the dye concentration was 0.3 to 1.0 (o.w.f). After treatment, the dry rubbing fastness of dyed sample was improved by 0.5 level. The wet rubbing fastness was improved after treatment. These results show that the existence of oligomers has a negative impact on the fabric dyeing in the silicone waterless dyeing system.

### 3.4. SEM Analysis

**Figure 4:**
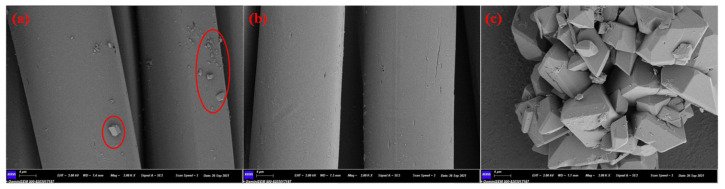
SEM images of original polyester (**a**), treated polyester (**b**) and oligomer (**c**).

The SEM micrographs of the surfaces of original polyester, treated polyester and oligomer are shown in Figure 4. As illustrated in Figure 4a, there are relatively more oligomers on the surface of untreated polyester. These oligomers will affect the uniform distribution of dyes. The presence of oligomers on the polyester (after treatment) is obviously reduced from the surface (Figure 4b). These observations support the results of the color uniformity tests. Moreover, the force between oligomers attached to the surface and polyester fiber is not firm. It can be predicted that the oligomer adsorbed on the surface of a certain dye will affect the color fastness of the dyed samples.

### 3.5. DSC Analysis

**Figure 5:**
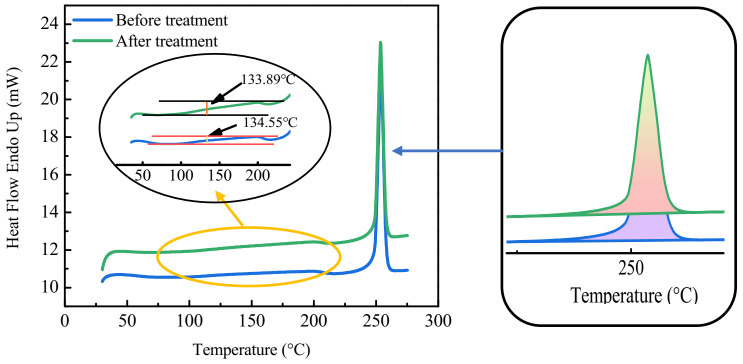
DSC curves of the polyester fabrics before and after treatment.

DSC analysis was carried out to determine the changes in the fabric after oligomer extraction. As can be seen from Figure 5, the DSC curves of polyester before and after oligomer extraction are considered to be similar. According to the obvious phase transition (glass transition) stage in the DSC curve, Tg (glass transition temperature) of the fabric changed from 134.55 °C to 133.89 °C [25,26]. Furthermore, corresponding to the DSC scanning information of the heating process, the melting process of the treated polyester sample is almost constant. The melting enthalpy can be obtained by integrating the melting peak. ΔH of the fabric changed from 60.36 J/g to 56.80 J/g after oligomer extraction. Since ΔH_0_ (the melting enthalpy of crystalline polyester) was 140 J/g [27], the crystallinity of the fabric could be changed from 43.11% to 40.57% [28]. This may also have been due to the removal of oligomers from the amorphous region, which affects the overall melting process [29]. The reason is that the existence of oligomers has some effects on the enthalpy change of PET melting and crystallization [30].

### 3.6. DMA Analysis

**Figure 6:**
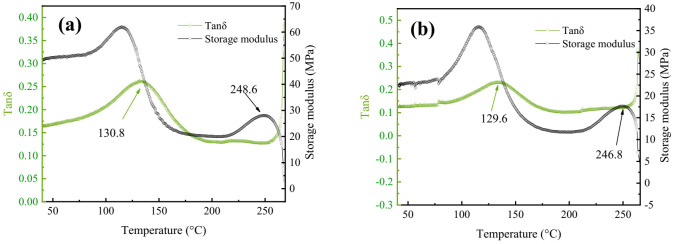
DMA curves of the polyester fabrics before (**a**) and after treatment (**b**): E’ (storage modulus) and tan δ (damping factor) are used as the main evaluation indicators here.

DMA of polyester samples selected for dyeing before and after solvent extraction of oligomers were measured and the results are shown in Figure 6. The test data of polyester samples were mainly analyzed from E’ (storage modulus) and tan δ (damping factor) to evaluate the fabric samples [31,32]. These two indexes can help judge Tg (glass transition temperature) and Tm (molten transition temperature) [33]. According to the test results, it can be seen that the storage modulus E’ and loss modulus E’ changed very little, indicating that the loss of elastic behavior and viscous behavior of polyester was very small after solvent extraction. As seen in Figure 6a, the glass transition temperature (Tg = 130.8 °C) and molten transition temperature (Tm = 248.6 °C) of polyester samples can be identified. According to the analysis of Figure 6b, the glass transition temperature of polyester after oligomer revising (Tg = 129.6 °C) had little change. It should be noted that the removal of some components (oligomers) may also affect the overall Tg [24]. It was verified that the mobility of polyester macromolecular segments was hardly changed after oligomer revision. Concurrently, the peak of storage modulus values showed that the melting point of the fabric decreased by only 2 °C (from 248.6 °C to 246.8 °C). Comparing the storage modulus of polyester fiber before and after treatment, it can be seen that there was almost no change, which proves that the elastic deformation part and plastic deformation part did not change, and the link of macromolecular segments did not deteriorate.

### 3.7. XRD Analysis

**Figure 7:**
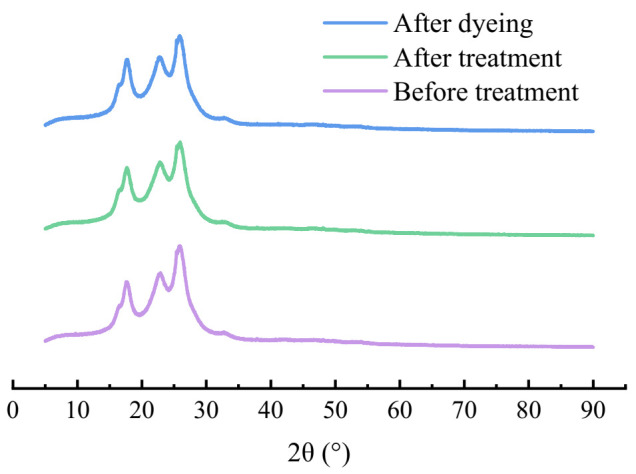
XRD spectra of the polyester fabrics before and after extraction (dyed sample was also used as a reference).

To study the effect of extracted oligomers on the crystallization properties of polyester, the XRD spectra of polyester before and after extraction were compared [34]. As shown in Figure 7, the diffraction curves of the original, after extraction and dyed fabrics were very similar, and the positions and shapes of each diffraction peak were basically the same. Even after dyeing in the waterless dyeing system, the XRD peaks of polyester samples were still the same. Under the same test conditions, the half-height width of each diffraction peak was very close. It indicates that the grain size and plane spacing of these samples have not changed remarkably. This result can prove that the crystallinity of the treated polyester sample is still very stable. Even after dyeing in medium, the crystal structure of polyester hardly changes.

## 4. Conclusions

In this research, the oligomers on the surface of polyester samples were extracted with solvent, and the effect of oligomers on polyester dyeing in the silicone waterless dyeing system was studied. Cyclic oligomers can be precipitated from polyester fiber in silicone waterless dyeing conditions. The content of the oligomers on polyester fiber was generally about 2%, which was close to the proportions and amounts of cyclic oligomers found in commercial polyester. After oligomer extraction, the color depth of dyed PET fabric was increased by 6–13%, accompanied by an increase of 3.25–3.71%. in dye exhaustion. Moreover, the concerned color uniformity and rubbing colorfastness of dyed samples were also improved. From the results of DSC, DMA and XRD of fabrics before and after extraction, the thermal and crystalline properties of polyester fiber were not influenced by the solvent treatment. Therefore, removing (or reducing) oligomers in polyester fabric is an effective way to improve polyester dyeing properties in silicone waterless dyeing systems. Moreover, the control of oligomers has been shown in this study to improve the levelness of dyed polyester fabrics. The quality of dyeing products may be greatly improved. Therefore, this study has guiding significance for the research direction of developing silicone waterless dyeing systems.

## Figures and Tables

**Table 1:** Gradient elution system for oligomer analysis.

Retention Time (min)	A% (Acetonitrile)	B% (Water)
0	30	70
15	100	0
20	100	0
21	30	70

**Table 2:** Oligomer contents of different polyester samples.

Polyester Sample	Oligomer Content	Standard Deviation
Fabric	2.15%	0.578%
Filament, 188D	2.05%	0.432%
Filament, 160D	2.03%	0.378%
Spun, 225D	1.65%	0.063%
Spun, 135D	1.18%	0.476%

**Table 3:** Effect of existence of oligomers on colorfastness properties of fabric dyed in waterless dyeing system.

Dye Concentration (% o.w.f.)	Before Treatment		After Treatment	
	Dry Rubbing Fastness	Wet Rubbing Fastness	Dry Rubbing Fastness	Wet Rubbing Fastness
0.3	4/5	5	5	5
0.5	4	4/5	4/5	5
0.7	3/4	4	4	4
1.0	2–3	3	3	3–4

## Data Availability

The raw/processed data required to reproduce these findings cannot be shared at this time as the data also forms part of an ongoing study.
